# Supplementary material for: Clinical Characteristics and Survival of Ovarian Cancer Patients According to Homologous Recombination Deficiency Status
Source: Cancers (Basel). 2025 May 12;17(10):1628. doi: 10.3390/cancers17101628 (PMC12109656; doi:10.3390/cancers17101628)
Supplement: Supplementary file 1 [file cancers-17-01628-s001.zip › cancers-3608358-supplementary.pdf]

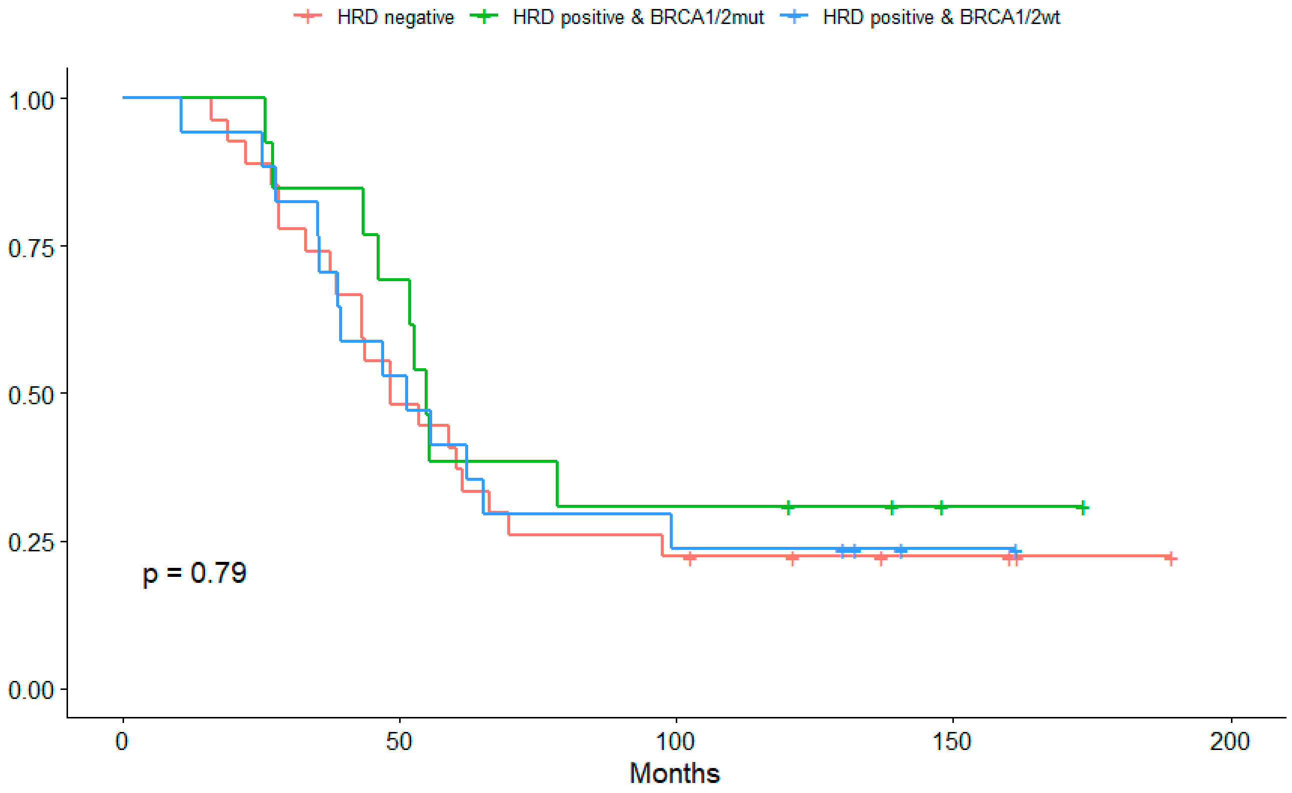

Figure S1. Overall survival with in-house GIS. Kaplan-Meier survival curves comparing HRD-negative patients, HRD-positive patients with *BRCA1/2* mutations, and HRD-positive patients without *BRCA1/2* mutations. The y-axis represent the estimated survival probability, while the x-axis shows time in months. The p-value of 0.79 is derived from the log-rank test, indicating no statistically significant difference in survival between the three groups. GIS: genomic instability score, HRD: homologous recombination deficiency.

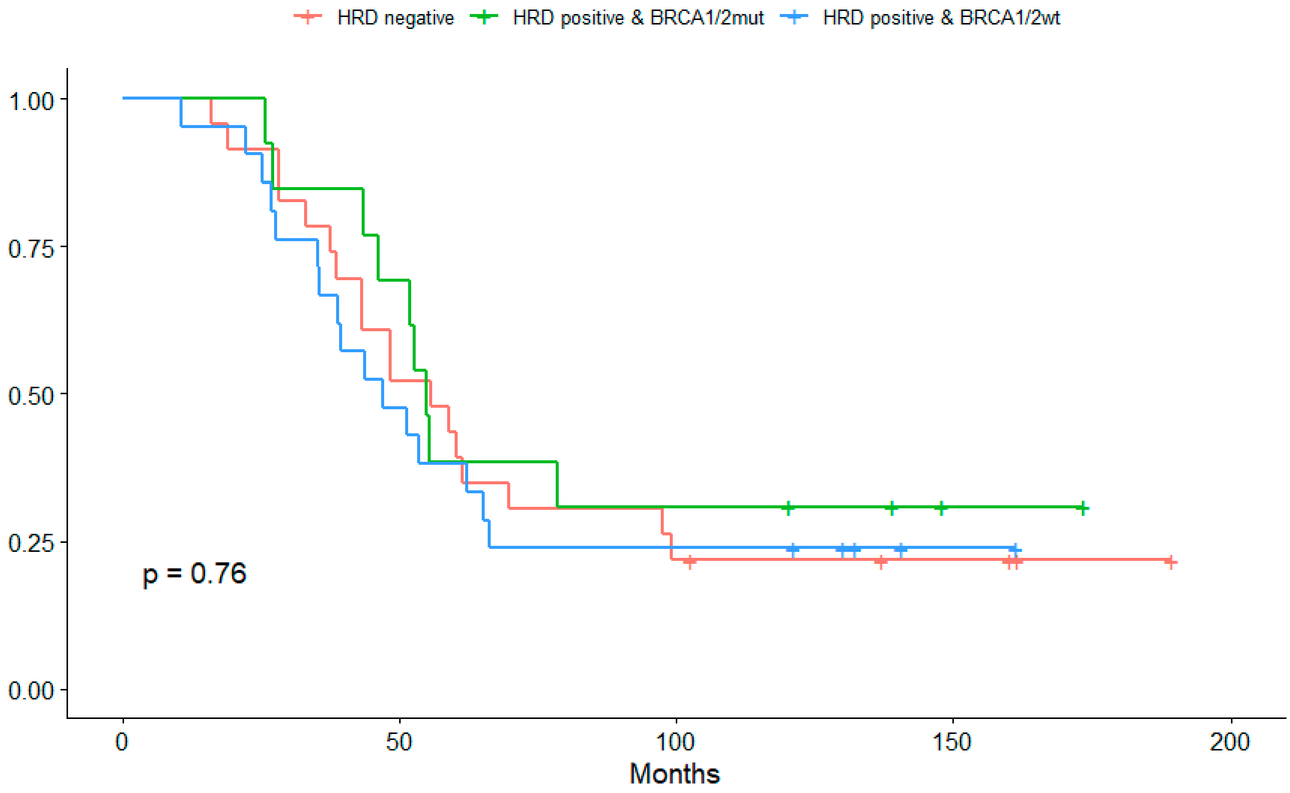

Figure S2. Overall survival with nLST. Kaplan-Meier survival curves comparing HRD-negative patients, HRD-positive patients with *BRCA1/2* mutations, and HRD-positive patients without *BRCA1/2* mutations. The y-axis represent the estimated survival probability, while the x-axis shows time in months. The p-value of 0.76 is derived from the log-rank test, indicating no statistically significant difference in survival between the three groups. HRD: homologous recombination deficiency, nLST: normalized large-scale transition score.

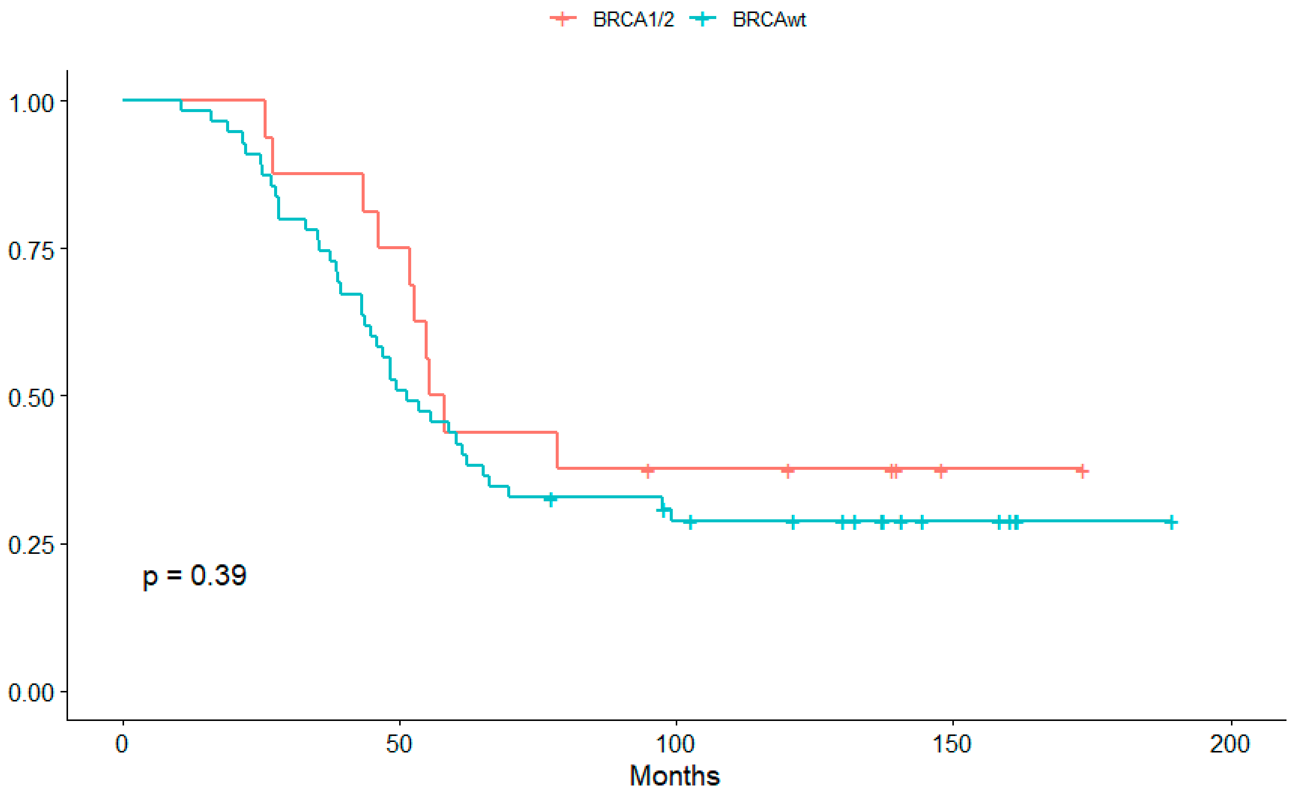

Figure S3. Kaplan-Meier overall survival curves comparing *BRCA1/2* mutated patients with *BRCAwt* patients. The y-axis represents the estimated survival probability, while the x-axis shows time in months. The p-value of 0.39 is derived from the log-rank test, indicating no statistically significant difference in survival between the groups.

Table S1. Clinical characteristics and survival of patients with discordant and concordant HRD status

	Discordant HRD (N=12)	Concordant HRD (N=59)	p-value
Age in years median (range)	67 (54-85)	63 (41-87)	0.101
CA125 median (range)	441 (96-4541)	462 (30-17160)	0.878
RMI median (range)	2444 (864-40869)	5082 (63-65070)	0.545
BMI median (range)	23 (19-35)	25 (19-41)	0.500
Follow up time in months median (range)	95 (63-123)	87 (65-123)	0.753
**Performance score**			
0-1	9 (75%)	54 (91%)	0.488
≥ 2	3 (25%)	5 (9%)	1.000
**FIGO stage**			
I-II	4 (34%)	10 (17%)	1.000
III-IV	8 (66%)	49 (83%)	1.000
**Residual tumor after surgery**			
0 - < 0	10 (83%)	39 (66%)	1.000
≥ 1	2 (17%)	20 (34%)	1.000
**Platinum response**			
> 12 months (sensitive)	6 (50%)	25 (42%)	0.870
6 - ≤ 12 months (partial sensitive)	6 (50%)	34 (34%)	0.870
**Survival in months**			
OS median (range)	55 (22-158)	55 (11-189)	0.707
PFS median (range)	18 (12-92)	22 (8-107)	0.465

Platinum sensitivity is defined as the absence of relapse or progressive disease withing 12 months after completing first-line platinum-based chemotherapy, while partially platinum sensitivity is defined as relapse occurring between 6 and 12 months after treatment completion. FIGO: International Federation of Gynecology and Obstetrics, HRD: homologous recombination deficiency, GIS: Genomic instability score, OS: overall survival, PFS: progression free survival, RMI: risk of malignancy index.

Table S2. Genes with pathogenic or likely pathogenic mutations in platinum-sensitive patients according to HRD status

**HRD status measured with GIS**				
**HRD positive (n=37)**				
ARID1A (n=1)	*CDK12 (n=1)*	*FGFR4 (n=1)*	*MSH2 (n=3)*	*PTCH1 (n=2)*
ATM (n=1)	*CREBBP (n=5)*	*FLT3 (n=1)*	*NOTCH1 (n=2)*	*SMARCA4 (n=1)*
BRCA1 (n=13)	*EGFR (n=1)*	*H3F3A (n=1)*	*PDGFRB (n=1)*	*TP53 (n=34)*
BRCA2 (n=3)	*FANCA (n=2)*	*KRAS (n=1)*	*PIK3CB (n=1)*	*TSC1 (n=2)*
**HRD negative (n=34)**				
ARID1A (n=1)	*KRAS (n=1)*	*NOTCH3 (n=2)*	*RAD51 (n=1)*	*SMO (n=1)*
CREBBP (n=1)	*NF1 (n=5)*	*PIK3CA (n=1)*	*RB1 (n=1)*	*TP53(n=29)*
FANCA (n=1)	*NOTCH1 (n=2)*	*POLE (n=1)*	*SMARCB1 (n=1)*	
**HRD status measured with nLST score**				
**HRD positive (n=43)**				
ARID1A (n=2)	*CREBBP (n=5)*	*H3F3A (n=1)*	*NOTCH3 (n=2)*	*SMARCA4 (n=1)*
ATM (n=1)	*EGFR (n=1)*	*KRAS (n=1)*	*PDGFRB (n=1)*	*SMARCB1 (n=1)*
BRCA1 (n=13)	*FANCA (n=1)*	*MSH2 (n=3)*	*PIK3CB (n=1)*	*SMO (n=1)*
BRCA2 (n=3)	*FGFR4(n=1)*	*NF1 (n=1)*	*POLE (n=1)*	*TP53 (n=39)*
CDK12 (n=1)	*FLT3 (n=1)*	*NOTCH1*	*PTCH1 (n=2)*	*TSC1 (n=2)*
**HRD negative (n=28)**				
CREBBP (n=1)	*KRAS (n=1)*	*NOTCH1*	*RAD51 (n=1)*	*TP53 (n=24)*
FANCA (n=1)	*NF1 (n=4)*	*PIK3CA (n=1)*	*RB1 (n=1)*	

The pathogenicity of the genes was defined by ClinVar and classified according to ACMG criteria.

Table S3. HRD status in 6 platinum-resistant patients with HRR gene mutations

HRR gene	HRD status	GIS	nLST	OS
BRCA1	Positive	72	40	55
BRCA1	Positive	59	16	28
BRCA2	Positive	65	27	11
CHEK1	Negative	3	0	48
CDK12	Positive	78	31	180
ATM	Negative	20	7	10

HRD positivity was determined using a cut-off of in-house GIS ≥ 50 and nLST ≥ 15. HRD: Homologous recombination deficiency, HRR: Homologous recombination repair, GIS: Genomic instability score, OS: overall survival.
